# Thromboelastometric Analysis of the Correlation Between Burn-Induced Coagulopathy and Severity of Burn Injury

**DOI:** 10.7759/cureus.54489

**Published:** 2024-02-19

**Authors:** Hiroyuki Koami, Yuichiro Sakamoto, Ayaka Matsuoka, Kota Shinada

**Affiliations:** 1 Department of Emergency and Critical Care Medicine, Saga University, Saga, JPN; 2 Department of Emergency and Critical Care Medicine, Saga University Hospital, Saga, JPN

**Keywords:** fibrinogen, extrinsic coagulation cascade, burn index, clot firmness, viscoelastic device

## Abstract

Background

In this study, we aimed to analyze the association between the burn index (BI) and burn-induced coagulopathy.

Methods

Adult burn patients transported to our emergency department who underwent rotational thromboelastometry (ROTEM) between April 1, 2013, and December 31, 2021, were enrolled in this study. The patients were categorized into two groups based on burn severity. Severe burns were defined as BI scores of > 15. Patient demographics, clinical variables of burns, standard laboratory test data, ROTEM data, and clinical outcomes of both groups were evaluated. In addition, the correlation between severe burns and significant variables was evaluated using a univariate analysis.

Results

Seven patients were enrolled and categorized into the severe (n = 2) and control (n = 5) groups. The severe group had a significantly worse consciousness level and higher mortality rate and showed higher tendencies of burn severity and clinical severity scores. Disseminated intravascular coagulation was confirmed in one patient. All ROTEM variables in the severe group regarding clot firmness in the extrinsic coagulation cascade (EXTEM) and fibrinogen-specific coagulation cascade (FIBTEM) showed a decreasing tendency as compared to those in the control group. Moreover, correlation analyses revealed strong correlations between the BI and clot firmness (rho = −0.946 to −0.721).

Conclusions

Severe BI was strongly associated with decreased blood clot firmness in EXTEM, FIBTEM, and ROTEM. Future research using viscoelastic devices may provide new possibilities for the treatment of severe burns.

## Introduction

Despite recent advancements in burn resuscitation and critical care, burn injuries remain a global public health concern [1]. Extensive burns are characterized by thermal stimuli-induced systemic inflammation and hypercoagulability, resulting in circulatory disturbances in multiple vital organs and, ultimately, poor clinical outcomes [2,3]. To date, little is known regarding the detailed mechanisms by which extensive thermal stimulation results in hypercoagulability.

Conventional coagulation markers, such as prothrombin time (PT) and activated partial thromboplastin time (APTT), are measured based on plasma, reflecting limited information on coagulation and fibrinolytic processes, excluding the contribution of platelets, which play a vital role in this complicated system [4]. In 2013, rotational thromboelastometry (ROTEM Delta), a viscoelastic device (VED) for assessing whole-blood profiles, was installed in the emergency department (ED). Coagulation and fibrinolytic abnormalities were evaluated as point-of-care in every emergency patient admitted to the ED and intensive care unit. ROTEM demonstrates the characteristic cascades of the comprehensive coagulation and fibrinolytic system, including the extrinsic coagulation cascade (EXTEM), intrinsic coagulation cascade, fibrinogen-specific coagulation cascade (FIBTEM), and anti-fibrinolytic cascades (APTEM) [5]. An increasing body of evidence supports the usefulness of such VED in trauma resuscitation. However, few studies have evaluated coagulopathy using VED during the acute phase in patients with burns [6].

In this study, we proposed the hypothesis that burn area-dependent systemic inflammation could correlate with the degree of coagulation abnormalities, even when patients with burns are transported to the ED. We analyzed the association between the burn area and burn-induced coagulopathy based on VED data and other parameters retrospectively collected from the patient charts.

## Materials and methods

This single-center retrospective study was approved by the institutional review board of Saga University Hospital (approval number: 2022-04-R-03) and was conducted in accordance with the tenets of the Declaration of Helsinki. Burn patients aged > 18 years who were transported to our hospital by ambulance and underwent ROTEM in the ED between April 1, 2013, and December 31, 2021, were enrolled in this study. In addition, we only considered the patients’ first blood tests upon admission to our hospital to exclude the influence of other treatments. Patients with single airway burns and those transferred from other hospitals were excluded from this study. Moreover, all patients were given an opportunity to opt out of the study if desired.

Patient information, clinical data on burns, blood test data at presentation, ROTEM data, and clinical outcomes were collected from the electronic medical records. Severe burns were defined as a burn index (BI) > 15 [7]. The enrolled patients were categorized into two groups based on the severity of the burn injuries. Furthermore, burn severity correlated with the extent of burn-induced coagulation abnormalities. The variables analyzed in this study included sex, age, presence of flame burns, BI, prognostic BI (PBI) [7], presence of airway burns, severity according to Artz’s criteria [8], heart rate, and Glasgow Coma Scale (GCS) score. These variables included body temperature, systemic inflammatory response syndrome score, disseminated intravascular coagulation (DIC) criteria from the Japanese Association for Acute Medicine (JAAM) [9]; and Acute Physiology and Chronic Health Evaluation (APACHE) II score. Sequential organ failure assessment (SOFA) score; length of hospital stay; mortality rate; white blood cell and platelet count; C-reactive protein, hemoglobin, fibrinogen, PT-international ratio, APTT, fibrin-fibrinogen degradation products (FDP), D-dimer (DD), creatine kinase, and lactate levels; pH; and base excess (BE) were also analyzed. In addition, the variables analyzed through ROTEM included clotting time (CT), clot formation time, alpha angle (α), amplitude “X” minutes after CT (A “X”), maximum clot firmness (MCF), lysis index “X” minutes after CT (LI “X”) in EXTEM, FIBTEM, and APTEM. Fibrinolysis shutdown was defined as LI60 > 96.5 for EXTEM, following previous reports [10]. Finally, a univariate analysis was performed for significant factors, and all patients were categorized into two groups with a median EXTEM MCF of 66.

Statistical analysis was performed using JMP®Pro 16.1.0 (SAS Institute Inc., NC, 1989-2021). In the univariate analysis, the Wilcoxon test was used for analyzing continuous variables, expressed as medians (interquartile range; Q1, Q3), whereas the chi-square test and Fisher’s exact test were used for analyzing nominal variables, expressed as numbers (%). Spearman’s rank correlation coefficient was used for the correlation analysis. Statistical significance was set at P < 0.05.

## Results

Seven patients were enrolled in this study and categorized into severe (n = 2) and control (n = 5) groups. The median age of the patients was 73 years; six males (85%) had a median BI of 9.1 and two had a BI > 15 (Table 1). Among the seven patients, DIC was confirmed in one patient, and two patients (28.6%) died during the study period.

**Table 1:** Characteristics of all enrolled patients BI, burn index; PBI, prognostic BI; JAAM DIC, Japanese Association for Acute Medicine disseminated intravascular coagulation; APACHE, Acute Physiologic Assessment and Chronic Health Evaluation; SOFA, Sequential Organ Failure Assessment; LOS, length of stay

Variables	Data
Age, y/o	73 (30, 85)
Male sex, n (%)	6 (85)
Flame burn, n (%)	6 (85)
BI	9.1 (8.0, 46.5)
BI > 15, n (%)	2 (28.6)
PBI	93 (58, 124)
Airway burn, n (%)	2 (28.6)
Artz’s criteria (severe), n (%)	3 (42.9)
JAAM DIC, n (%)	1 (14.3)
APACHE II	11 (11, 17)
SOFA_total	1 (0, 6)
LOS, days	31 (3, 54)
Mortality rate, n (%)	2 (28.6)

Patient characteristics were similar in both groups (Table 2). The severe group not only had a significantly worse level of consciousness (GCS, 7 vs. 15, P = 0.030) and a higher mortality rate (100% vs. 0%, P = 0.048) but also showed a trend towards higher burn and clinical severity scores. Other variables, including the JAAM DIC (Japanese Association for Acute Medicine disseminated intravascular coagulation) score, were statistically similar between the groups.

**Table 2:** Patient characteristics, data on burn, vital signs, clinical scores, and outcomes BI, burn index; PBI, prognostic BI; GCS, Glasgow Coma Scale; SIRS, Systemic Inflammatory Response Syndrome; JAAM DIC, Japanese Association for Acute Medicine disseminated intravascular coagulation; APACHE, Acute Physiologic Assessment and Chronic Health Evaluation; SOFA, Sequential Organ Failure Assessment; LOS, length of stay

	Severe group (n = 2)	Control group (n = 5)	P value (< 0.05)
Age, y/o	60 (30, 90)	73 (39, 83)	0.85
Male sex, n (%)	2 (100)	4 (80)	1.00
Flame burn, n (%)	1 (50)	5 (100)	0.29
BI	70.3 (46.5, 94.0)	9.0 (4.8, 11.6)	0.08
PBI	130 (124, 137)	82 (44, 94)	0.08
Airway burn, n (%)	1 (50)	1 (20)	1.00
Artz’s criteria (severe), n (%)	2 (100)	1 (20)	0.14
Heart rate, bpm	141 (120, 161)	100 (78, 116)	0.18
GCS	7 (6, 7)	15 (15, 15)	0.030
Body temperature, ℃	35.4 (33.0, 37.8)	36.8 (36.6, 37.4)	1.00
SIRS, pts	3 (2, 3)	1 (1, 3)	0.31
JAAM DIC, pts	3 (1, 4)	0 (0, 3)	0.32
JAAM DIC, n (%)	1 (50)	0 (0)	0.29
APACHE II	20 (17, 23)	11 (6, 12)	0.07
SOFA_total	7 (6, 7)	0 (0, 1)	0.07
LOS, days	3 (3, 3)	36 (17, 65)	0.33
Mortality rate, n (%)	2 (100)	0 (0)	0.048

Although BE showed a decreasing tendency in the severe group, blood test findings were statistically comparable with respect to other parameters, including those of standard coagulation tests (Table 3).

**Table 3:** Blood testing profile WBC, white blood cell; CRP, C-reactive protein; Hb, hemoglobin; Plt, platelet count; Fib, fibrinogen; PT-INR, prothrombin time-international normalized ratio; APTT, activated partial thromboplastin time; FDP, fibrin/fibrinogen degradation products; DD, D-dimer; CK, creatine kinase; BE, base excess; Lac, lactate

	Severe group (n = 2)	Control group (n = 5)	P value (< 0.05)
WBC, /µL	11600 (9300, 13900)	14500 (10850, 22700)	0.56
CRP, mg/dL	0.13 (0.01, 0.24)	0.08 (0.03, 0.14)	1.00
Hb, g/dL	16.7 (15.3, 18.0)	14.6 (14.3, 15.6)	0.18
Plt, ×10^4^/µL	29.3 (15.5, 43.1)	27.0 (19.7, 29.4)	0.85
Fib, mg/dL	290 (223, 357)	356 (319, 502)	0.56
PT-INR	0.98 (0.89, 1.06)	1.00 (0.97, 1.00)	1.00
APTT, s	25.5 (22.3, 28.6)	28.3 (26.7, 37.0)	0.56
FDP, µg/mL	38.3 (17.3, 59.3)	18.7 (1.7, 25.8)	0.77
DD, µg/mL	12.6 (8.3, 17.0)	4.9 (0.7, 11.7)	0.25
CK, U/L	295 (207, 382)	218 (101, 1497)	1.00
pH	7.35 (7.25, 7.45)	7.39 (7.38, 7.46)	0.85
BE, mmol/L	-7.1 (-7.4, -6.7)	-0.6 (-3.0, 2.2)	0.08
Lac, mmol/L	5.3 (3.7, 6.9)	1.9 (1.5, 5.7)	0.33

The ROTEM data are presented in Table 4. All variables in the severe group regarding clot firmness, such as A5, A10, A30, and MCF in EXTEM and FIBTEM, showed a decreasing tendency compared to the control group. In addition, a lower tendency was observed in the severe group than in the control group. No significant differences in other ROTEM variables were observed between the groups.

**Table 4:** ROTEM data ROTEM, rotational thromboelastometry; EXTEM, extrinsic coagulation cascade; FIBTEM, fibrinogen-specific coagulation cascade; APTEM, anti-fibrinolytic cascades, CT, clotting time; CFT, clot formation time; α, alpha angle; AX, amplitude “X” minutes after CT; MCF, maximum clot firmness; LI 60, lysis index 60 minutes after CT; SD, fibrinolysis shutdown

		Severe group (n = 2)	Control group (n = 5)	P value (< 0.05)
EXTEM	CT, s	66 (61, 71)	60 (54, 74)	0.85
	CFT, s	102 (71, 132)	67 (66, 89)	0.33
	α, °	74 (72, 75)	76 (73, 78)	0.33
	A5, mm	41 (36, 46)	51 (50, 52)	0.08
	A10, mm	52 (48, 55)	61 (60, 64)	0.08
	A30, mm	61 (58, 63)	66 (66, 70)	0.07
	MCF, mm	61 (58, 63)	66 (66, 71)	0.07
	LI60, %	94 (92, 96)	92 (87, 96)	0.55
FIBTEM	A5, mm	9 (7, 10)	16 (14, 18)	0.08
	A10, mm	9 (7, 11)	18 (16, 19)	0.08
	A30, mm	10 (7, 12)	19 (17, 21)	0.08
	MCF, mm	10 (7, 12)	19 (17, 21)	0.08
	LI60, %	86 (84, 87)	99 (97, 100)	0.08
APTEM	LI60, %	96 (94, 98)	92 (88, 95)	0.18
SD, n (%)		0 (0.0)	1 (20.0)	1.00

Subsequently, we analyzed the correlations between the BI and the associated factors identified using univariate analysis, as well as those used in the standard coagulation test (Table 5). The APACHE II score (Rho = 0.852), SOFA score (0.730), FDP (0.700), DD (0.943), BE (−0.857), A5 (−0.946), A10 (−0.775), and MCF (−0.741) in EXTEM and A5 (−0.750) and A10 (−0.721) in FIBTEM showed strong correlation with BI.

**Table 5:** Correlation analyses compared to the burn index APACHE, Acute Physiologic Assessment and Chronic Health Evaluation; SOFA, sequential organ failure assessment; Plt, platelet count; Fib, fibrinogen; PT-INR, prothrombin time-international normalized ratio; FDP, fibrin/fibrinogen degradation products; DD, D-dimer; BE, base excess; E, EXTEM; AX, amplitude “X” minutes after clotting time (CT); MCF, maximum clot firmness; F, FIBTEM; LI 60, lysis index 60 minutes after CT

	Correlation coefficient (Rho)	P value (< 0.05)
APACHE II	0.852	0.01
SOFA_total	0.730	0.06
Plt, ×10^4^/µL	-0.179	0.70
Fib, mg/dL	-0.321	0.48
PT-INR	0.037	0.94
FDP, µg/mL	0.700	0.19
DD, µg/mL	0.943	0.005
BE, mmol/L	-0.857	0.01
E_A5, mm	-0.946	0.001
E_A10, mm	-0.775	0.041
E_MCF, mm	-0.741	0.06
F_A5, mm	-0.750	0.052
F_A10, mm	-0.721	0.07
F_MCF, mm	-0.685	0.09
F_LI60, %	-0.469	0.29

**Figure 1:**
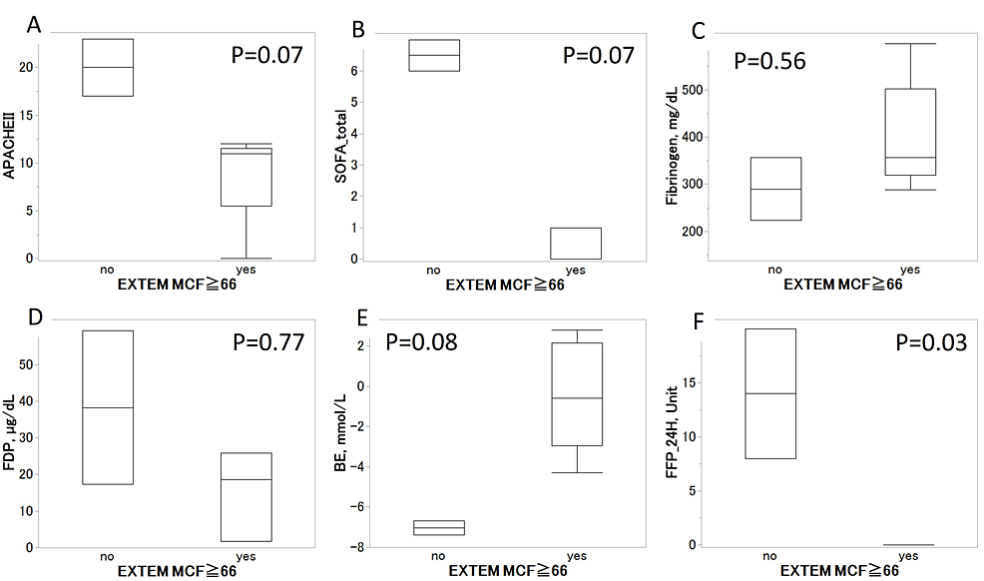
Association between MCF in EXTEM and clinical scores, coagulation markers, and BE and FFP transfusion The Wilcoxon test demonstrated higher tendencies of APACHE II (P = 0.07) and SOFA scores (P = 0.07) in the group without increased MCF in EXTEM and a lower tendency of BE (P = 0.08) in the same population. The transfusion volume of FFP in the non-high-MCF group was significantly higher than that in the high-MCF group (P = 0.03). There were no significant differences in the fibrinogen and FDP values. MCF, maximum clot firmness; EXTEM, extrinsic coagulation cascade; BE, base excess; fresh frozen plasma (FFP); APACHE, Acute Physiologic Assessment and Chronic Health Evaluation; SOFA, sequential organ failure assessment; FDP, fibrin/fibrinogen degradation products

Finally, we focused on the MCF in EXTEM and categorized the patients into two groups with a median of 66, evaluating the relationship between burn severity and variables that were significant in the previous analyses (Figure 1). Elevated APACHE II and SOFA scores and decreased BE were observed in patients without an MCF ≥ 66. However, no statistically significant differences were observed in the fibrinogen and DD values. The total volume of fresh frozen plasma (FFP) transfusions during the first 24 h after admission to the ED (14 U vs. 0 U, P = 0.030) and mortality rate (100% vs. 0%, P = 0.048, data not shown) were significantly higher in patients without increased MCF in EXTEM.

## Discussion

In the present study, patients with an elevated BI exhibited decreased clot firmness in ROTEM parameters at the time of admission to the ED. Furthermore, clot firmness in EXTEM and FIBTEM was strongly inversely correlated with BI, indicating that burn-induced hypercoagulability could reduce clot firmness in the extrinsic coagulation system, mainly involving fibrinogen.

The number of patients assessed in this study was extremely limited. Therefore, we evaluated the patient population in the critically ill group for outcome validity based on previous reports. We conducted a literature search on PubMed using search terms {(“burn” OR “thermal injury”) AND (“ROTEM” OR “thromboelastometry”)} to compare coagulation-fibrinolytic system markers, including ROTEM parameters, between the severe group in this study and previously reported severe burn patients. We identified only two studies that evaluated ROTEM parameters (Table 6) [11,12]. Both studies were prospective, with each including fewer than 20 cases. Although the definition of severe populations differs among studies, we suggest that the parameters of coagulation abnormalities in each patient group are comparable. It is particularly noteworthy that burn-related coagulation abnormalities develop immediately after burn injury, exemplified by severe cases presenting with burn-related DIC at the time of admission to the ED. The reported incidence of DIC due to thermal injury is highly variable, ranging from 0.09% and 91.1%. However, the exact frequency is unknown because all the reported studies had limited sample sizes [13,14]. Furthermore, previous studies have been reported that the mechanism of abnormal coagulation due to burn injury is not related to intravenous fluid administration but to direct vascular endothelial cell damage and tissue hypoperfusion, a tendency that was corroborated in this study [15].

**Table 6:** Comparisons between our data and previous data, which are analyzed using ROTEM BI, burn index; TBSA, total burn surface area; APACHE, Acute Physiologic Assessment and Chronic Health Evaluation; SOFA, sequential organ failure assessment; Plt, platelet; PT-INR, prothrombin time-international normalized ratio; APTT, activated partial thromboplastin time; E, EXTEM; CT, clotting time; CFT, clot formation time; MCF, maximum clot firmness; F, FIBTEM

	Our “Severe group”	Lonic et al., 2020 [11]	Schaden et al., 2012 [12]
Type of research	Retrospective	Prospective	Prospective
Definition	BI > 15	≥ 20% TBSA, III	> 15% TBSA, IIb-III
Number of patients	2	12	20
Age, y/o	60 (30, 90)	42 +- 15	53 +- 22
Male sex, n (%)	2 (100)	6 (50)	14 (70)
BI	70.3 (46.5, 94.0)	-	-
% TBSA burned	-	58.9 +-18.7	42.55 +- 21.81
APACHE II	20 (17, 23)	17.4 +- 5.8	-
SOFA	7 (6, 7)	9.4 +- 2.6	-
Plt, ×10^4^/µL	29.3 (15.5, 43.1)	27.59 +- 11.23	28.78 +- 18.96
PT-INR	0.98 (0.89, 1.06)	1.23 +- 0.18	-
APTT, s	25.5 (22.3, 28.6)	53.6 +- 16.9	38.7 +- 6.3
Fibrinogen, mg/dL	290 (223, 357)	198.6 +- 76.1	303.1 +- 120.1
E_CT, s	66 (61, 71)	48.7 +- 12.3	64.6 +- 18.8
E_CFT, s	102 (71, 132)	97.5 +- 32.1	99.8 +- 43.6
E_MCF, mm	61 (58, 63)	63.1 +- 5.9	60.4 +- 6.3
F_MCF, mm	10 (7, 12)	18.4 +- 7.5	17.2 +- 9.4
Mortality, n (%)	2 (100)	2 (16.7)	5 (25.0)

Coagulation and fibrinolytic abnormalities observed in the acute phase of thermal injury include a state of increased thrombogenicity related to hypercoagulation, relative inhibition of anticoagulability, and suppression of fibrinolysis. These are known to change dynamically based on the duration of burn stimuli, burn severity, and past medical conditions [2]. A previous report by Ball et al. suggested that platelets do not show considerable changes, whereas coagulation markers, such as fibrinogen, DD, and thrombin-antithrombin complex (TAT), increase during the acute phase. In addition, although prolonged PT and APTT, low platelet counts, low fibrinogen levels, and a significant increase in DD have been observed when DIC is diagnosed, no reliable single marker has been identified to date for the diagnosis of DIC [16]. No significant variables associated with severe burn injuries were identified in this study, although we observed increased clot formation at lower fibrinogen levels and higher DD levels. These findings are consistent with those of Lin et al.’s study involving non-survival cases [17].

In this study, ROTEM provided novel evidence that has not been previously recognized during practice in the ED. The decrease in clot firmness in EXTEM and FIBTEM after severe burns was unexpected compared to the hypercoagulable state induced by burn injuries. Considering that clot firmness in these assays is strongly correlated with BI, a consistent decrease in clot firmness, which is inversely dependent on burn severity, appears to be an essential finding. Since it is generally thought that blood clot firmness reflects platelet and fibrinogen values and their function, it is possible that these changes may reflect the consumption of fibrinogen and platelets with increased thrombogenesis [5]. For instance, fibrinogen is known to drop to critical levels early in the acute phase of severe trauma, indicating that a similar mechanism may occur during the acute phase of severe burn [18]. Moreover, platelet aggregation capacity is dependent on fibrinogen levels. Therefore, early, severe burn-induced fibrinogen depletion may result in impaired platelet aggregation [19]. We suggest that a decrease in fibrinogen levels and accompanying impaired platelet aggregation may accelerate the reduction in blood clot firmness.

This study has several limitations. The small sample size is a major factor. In addition, retrospective and single-center studies require caution when interpreting the data. Selection bias must be considered because ROTEM was measured only when physicians who could operate the ROTEM were shifted to the ED.

## Conclusions

In this retrospective study, patients with an elevated BI experienced reduced clot firmness in ROTEM parameters upon admission to the ED. Furthermore, this study highlighted the potential implications of burn injuries on coagulation parameters in a critical care setting. The results also showed a strong inverse correlation between BI and clot firmness in EXTEM and FIBTEM. These findings suggest that the burn-induced hyperinflammatory response and hypercoagulability could negatively impact the extrinsic coagulation system, particularly fibrinogen.

Although it is unclear whether impaired clot firmness leads to bleeding during the resuscitation phase of severe burns, further research using viscoelastic devices may offer new possibilities for treating severe burns.
